# Association between genetically predicted expression of *TPMT* and azathioprine adverse events

**DOI:** 10.1186/s40360-026-01093-4

**Published:** 2026-02-07

**Authors:** Alyssa Steitz, Laura L. Daniel, Puran Nepal, Alyson L. Dickson, Jacy Zanussi, Tyne W. Miller-Fleming, Peter S. Straub, Wei-Qi Wei, Ge Liu, Jennifer Maizel, Nancy J. Cox, Adriana M. Hung, QiPing Feng, C. Michael Stein, Cecilia P. Chung

**Affiliations:** 1 Department of Medicine, Vanderbilt University Medical Center, Nashville, USA; 2 Department of Medicine, University of Miami, Miami, USA; 3 Vanderbilt Genetics Institute, Vanderbilt University School of Medicine, Nashville, USA; 4 Department of Biomedical Informatics, Vanderbilt University Medical Center, Nashville, USA

**Keywords:** Azathioprine, Leukopenia, PrediXcan, Pharmacogenomics, Pharmacogenetics, Personalized medicine

## Abstract

**Background:**

Polymorphisms *thiopurine-S-methyltransferase* (*TPMT*) and *nudix hydrolase 15* (*NUDT15*) can increase the risk of azathioprine myelotoxicity, but little is known about other genetic factors that increase risk for azathioprine-associated side effects. PrediXcan is a gene-based association method that estimates the expression of individuals’ genes and examines their correlation to specified phenotypes. To interrogate the utility of using PrediXcan as a tool for detecting associations between genetic factors and azathioprine side effects, we aimed to determine whether the genetically predicted expression of *TPMT* or *NUDT15* was associated with leukopenia or other known side effects of azathioprine.

**Methods:**

This is a retrospective cohort study of 1,364 new users of azathioprine (patients taking azathioprine with an organ transplant indication were excluded) with electronic health record (EHR)-reported White race. We used PrediXcan to impute expression in liver tissue, tested its association with pre-specified phecodes representing known side effects (e.g. skin cancer), and completed chart review to confirm cases.

**Results:**

Among confirmed cases, patients in the lowest tertile of *TPMT* predicted expression had significantly higher odds of developing leukopenia (OR = 3.30, 95%CI: 1.07–10.20, *p* = 0.04) versus those in the highest tertile; no other side effects were significant.

**Conclusion:**

The results suggest that this methodology could be deployed on a larger scale to uncover associations between genetic factors and drug side effects for more personalized care.

**Supplementary Information:**

The online version contains supplementary material available at 10.1186/s40360-026-01093-4.

## Background

Azathioprine is an immunosuppressive drug used to treat a variety of inflammatory conditions, including systemic lupus erythematosus (SLE), systemic vasculitis, and inflammatory bowel disease (IBD). The use of azathioprine is frequently constrained by side effects, for which a limited number of genetic predictors are known. [1] One such example is dose-dependent myelotoxicity, a common and serious side effect of azathioprine use. [2, 3] At the start of azathioprine metabolism, it is rapidly converted to 6-mercaptopurine and then transported inside the cell. Through a series of enzymatic reactions, the drug is converted into its active metabolite, 6-thioguanine nucleotides (6-TGN). This metabolite suppresses the immune system by incorporating into DNA and RNA. However, too much 6-TGN is toxic and can cause bone marrow suppression. *Thiopurine S methyltransferase (TPMT)* can inactivate 6-TGN; therefore, alteration to *TPMT* expression will affect therapeutic dose. *Nudix hydrolase 15* (*NUDT15)* can cause myelosuppression through a similar mechanism that allows for a buildup of toxic azathioprine metabolites. [4] The *TPMT* findings, along with more recent findings regarding associations between variants in *NUDT15* and azathioprine-induced myelotoxicity are now well established and have been incorporated into Clinical Pharmacogenetics Implementation Consortium (CPIC) guidelines for azathioprine dosing. [5] However, many cases of myelotoxicity occur in patients with normal *TPMT* and *NUDT15* metabolism [6]; moreover, patients experience numerous additional side effects that can limit azathioprine use and burden patients, including rashes, malignancies, gastrointestinal intolerance, and infections. [7–10] While genome-wide association studies (GWAS) have identified some of the genetic predictors of leukopenia [5], little is known about how emerging methods in pharmacogenetics can add to our knowledge of genetic factors that increase risk for other azathioprine-associated side effects.

PrediXcan is a technique that estimates gene expression determined by an individual’s genetic profile through use of reference transcriptome data sets. [11] Given that PrediXcan estimates genetic expression by incorporating information from multiple single nucleotide polymorphisms (SNPs) in a biologically relevant way, this approach may potentially provide additional insights and increased power compared to assessments of SNPs as individual variables; as such, PrediXcan offers the possibility of identifying associations that have not been detectable previously using traditional GWAS or candidate SNP approaches. [11] To evaluate the feasibility of using PrediXcan to identify associations between genetic markers and side effects of azathioprine, we assessed whether the genetically predicted expression of *TPMT* or *NUDT15* was associated with leukopenia or other known side effects of azathioprine use.

## Methods

### Data collection

The cohort was derived from BioVU, a clinical practice-based biobank at Vanderbilt University Medical Center, a tertiary care center. BioVU uses de-identified electronic health records (EHRs)—including demographics, clinical notes, medical history, problem lists, medications, and diagnostic and procedure codes—which are linked with stored DNA samples. [12, 13] Informed consent to donate leftover blood samples from routine lab testing to BioVU for research was obtained from all subjects and/or their legal guardian(s).

For this retrospective cohort, we identified new users of azathioprine with at least one international classification of disease (ICD)-9 or ICD-10 code in their EHR during follow-up, as described below. We excluded patients whose genetic data did not pass quality control or whose primary indication for azathioprine was an organ transplant. Given that PrediXcan has been validated primarily in patients of European ancestry [14], we restricted the cohort to patients with EHR-reported White race; in previous work, we determined EHR-reported White race was highly concordant with European ancestry among new users of azathioprine in BioVU (i.e., > 99% of patients identified as having White race had predominantly European ancestry).[15]

Records review was completed to confirm azathioprine use and gather covariates. Patients entered the cohort on the first day of new azathioprine use, defined as no previous mention of azathioprine or mercaptopurine use in the EHR. Follow-up ended on the first of the following: 1) day of azathioprine discontinuation; 2) last confirmed azathioprine prescription or use as per the EHR plus 90 days; 3) lost to follow-up; 4) day of death; or 5) end of the study (December 31, 2018). We abstracted additional data from the EHR such as reported race, sex, age, initial daily dose of azathioprine, and indication.

As previously described [6], we genotyped patients using the Illumina Infinium Expanded Multi-Ethnic Genotyping Array (MEGA)^EX^ platform, imputed the results using Michigan imputation servers [16], and applied standard genetic quality controls. The Michigan Imputation Server estimates allele dosage by summing the posterior probabilities of having the alternate allele in the haplotype. After allele imputation, we used PrediXcan to estimate the expression of *TPMT* and *NUDT15* in liver tissue; we pre-specified liver tissue as the most appropriate target given that most of azathioprine metabolism is hepatic. [17] PrediXcan estimates genetic expression by combining genotype and MASHR (multivariate adaptive shrinkage in R) GTEx (Genotype Tissue Expression) weights (version 8). The MASHR weights set employed utilizes the MASH algorithm to perform SNP selection from variety of SNPs that might predict genetic expression. [18, 19] MASHR weights are tissue-specific and could vary by tissue. To determine the weights, the GTEx project collected tissue samples from hundreds of people and measured tissue expression of genes using RNA, then used the corresponding genetic data to predict the expression of genes with various tissues using a penalized regression model that selected the most predictive SNPs and gave weight to those SNPs. We applied the weights for each SNP to the genetic data from our dataset. Within our dataset, the MASHR model identified one SNP as a significant predictor of *TPMT* liver expression (rs2842941 C > G) and two SNPs as predictors of *NUDT15* liver expression (rs2031775 G > A, rs117508465 *T* > G). We also confirmed that the MASHR *TPMT* SNP was not already included among SNPs used in CPIC guidelines or in linkage disequilibrium.

Using clinical pharmacology databases (e.g., Micromedex), the U.S. Food and Drug Administration package insert for azathioprine, and peer-reviewed publications, we compiled a list of the nine most common types of adverse effects clearly associated with azathioprine use: infections, gastrointestinal intolerance, hematologic toxicity, dermatologic symptoms, skin malignancies, non-skin malignancies, hepatotoxicity, constitutional symptoms, and pulmonary symptoms. [20–22] We then identified 51 phecodes, a method for assigning phenotypes using ICD-9 and ICD-10 diagnosis codes in claims data, corresponding to these nine adverse event types (Table S1). [23] We gathered ICD-9 and ICD-10 diagnosis codes for each patient during follow-up and designated patients with ≥2 phecodes, occurring on non-consecutive days, as potential cases. After assessing whether any of these phecode groups were associated with the predicted *TPMT* or *NUDT15* liver expression (described further below), we reviewed EHRs for adverse event types that had a pre-specified *p* < 0.10, using clinical notes to confirm that the adverse effect could be reasonably attributed to azathioprine. We reviewed all potential cases for leukopenia, rash, and non-basal cell skin cancer. Leukopenia was defined as white blood cell count (WBC) < 4.0 K/μL, which was not attributed to other causes. Cases of rash were limited to those with an ICD code for rash that was not attributed to other causes (e.g., malar rash as a symptom of SLE). Skin cancer cases were biopsy-proven or reported in dermatology documentation. Review was completed by a clinician using a standardized adjudication form (Figure S1). [24, 25] Ambiguous cases were subject to final adjudication by the senior author. All adjudications were completed blinded to genotype and predicted expression. We did not review charts for potential cases not identified by a phecode.

### Statistical analysis

For demographic and clinical characteristics, we present categorical variables as numbers and percentages, and continuous variables as means and standardized differences. We used Chi-squared tests to compare categorical variables and Kruskal-Wallis tests for continuous variables.

To validate the predicted expression of *TPMT* and *NUDT15,* we compared it with the CPIC metabolizer phenotype using Kruskal-Wallis. The CPIC guidelines for *TPMT* and *NUDT15* make dosing recommendations to physicians prescribing a thiopurine based on metabolizer phenotype. The guidelines are based on a systematic literature review and compiled by an expert working group. The guidelines classify metabolizer status for *TPMT* and *NUDT15* as normal, indeterminate, intermediate, or poor based on genetic variants. As in our previous work, we grouped the metabolizer status as normal/indeterminate and intermediate/poor due to the relatively small numbers of patients classified as poor or indeterminate. [6] We next analyzed the association between predicted expression of *TPMT* and the 51 phecodes corresponding to the adverse effects of azathioprine using logistic regression. Phecodes associated with adverse events with a *p* < 0.10 were deemed “potential” associations and progressed to the next step of clinical review for case validation. We did not adjust for multiple comparisons when testing phecodes because this was an exploratory step, and the primary analysis was completed using chart-reviewed confirmed cases. Finally, we grouped the predicted expression of *TPMT* in liver tissue into tertiles and conducted logistic regression to assess the association between predicted expression and adverse events confirmed by review. The *TPMT* SNP's predicted expression largely corresponded to the tertiles (G/G, C/G, and C/C carriages for Tertiles 1, 2, and 3, respectively). Tertile 1 represented those with the lowest predicted expression, while Tertile 3 represented those with the highest predicted expression. We also completed logistic regressions adjusted by (1) age at baseline, (2) sex, and (3) initial dose of azathioprine for each of the phenotypes. We adjusted for the initial dose because azathioprine-induced leukopenia is dose-dependent.

We gathered laboratory results during azathioprine use, including WBC counts. We used it to interrogate the association between *TPMT* predicted expression and WBC count ≤3.0 K/μL. We also conducted a sensitivity analysis for WBC ≤4.0 K/μL, and we assessed the *TPMT* metabolizer status of confirmed leukopenia cases, as defined by current CPIC guidelines, to ascertain whether any identified cases were among normal metabolizers and could thus show additive clinical utility. Analyses were conducted using Stata version 17.0 [26] and R version 4.1.0.[27]

## Results

After review of medical records, we confirmed 5640 patients were using azathioprine and passed genetic quality control. After excluding patients with organ transplant and with a reported race that was not White, the cohort included 1364 patients (Figure S2). Patients were predominantly female (65.4%), their mean age was 44.7 ± 17.6 years old, and they had a mean follow-up time of 3.1 ± 3.8 years (min = 1 day, max = 22 years, median [IQR] = 1.59 years [0.26, 4.88]). Common indications for azathioprine included IBD (33.4%) and SLE (13.6%). There were no statistically significant differences in patient characteristics by tertile of *TPMT* predicted expression (Table 1).

**Table 1:** Baseline characteristics of new azathioprine users by tertile of predicted *TPMT* liver expression

	3 Tertiles of Estimated *TPMT* Liver Expression			p-value
	Tertile 1 (*N* = 455)	Tertile 2 (*N* = 456)	Tertile 3 (*N* = 453)	
Female, n (%)	290 (63.7)	299 (65.6)	303 (66.9)	0.61
Prioritized Indication, n (%)				0.14
Systemic Lupus Erythematosus	38 (8.4)	43 (9.4)	42 (9.3)	
Inflammatory Bowel Disease	145 (31.9)	148 (32.5)	162 (35.8)	
Other Inflammatory Disease	242 (53.2)	243 (53.3)	238 (52.5)	
Other	30 (6.6)	21 (4.6)	11 (2.4)	
Unknown	0 (0.0)	1 (0.2)	0 (0.0)	
Mean years of follow up, mean±SD	3.1±3.8	3.2±3.8	3.1±3.7	0.99
Mean age at initial dose, mean±SD	44.4±17.6	44.8±17.6	44.9±17.5	0.91
Initial Dose (in mg/day), mean±SD	74.9±46.2	81.1±50.5	77.4±47.4	0.17
Baseline WBC (in K/µL), mean±SD	8.8±3.7	8.8±3.8	8.8±3.6	0.98

Tertile 1 represents the lowest predicted expression; Tertile 3 represents the highest predicted expression

**Fig. 1:**
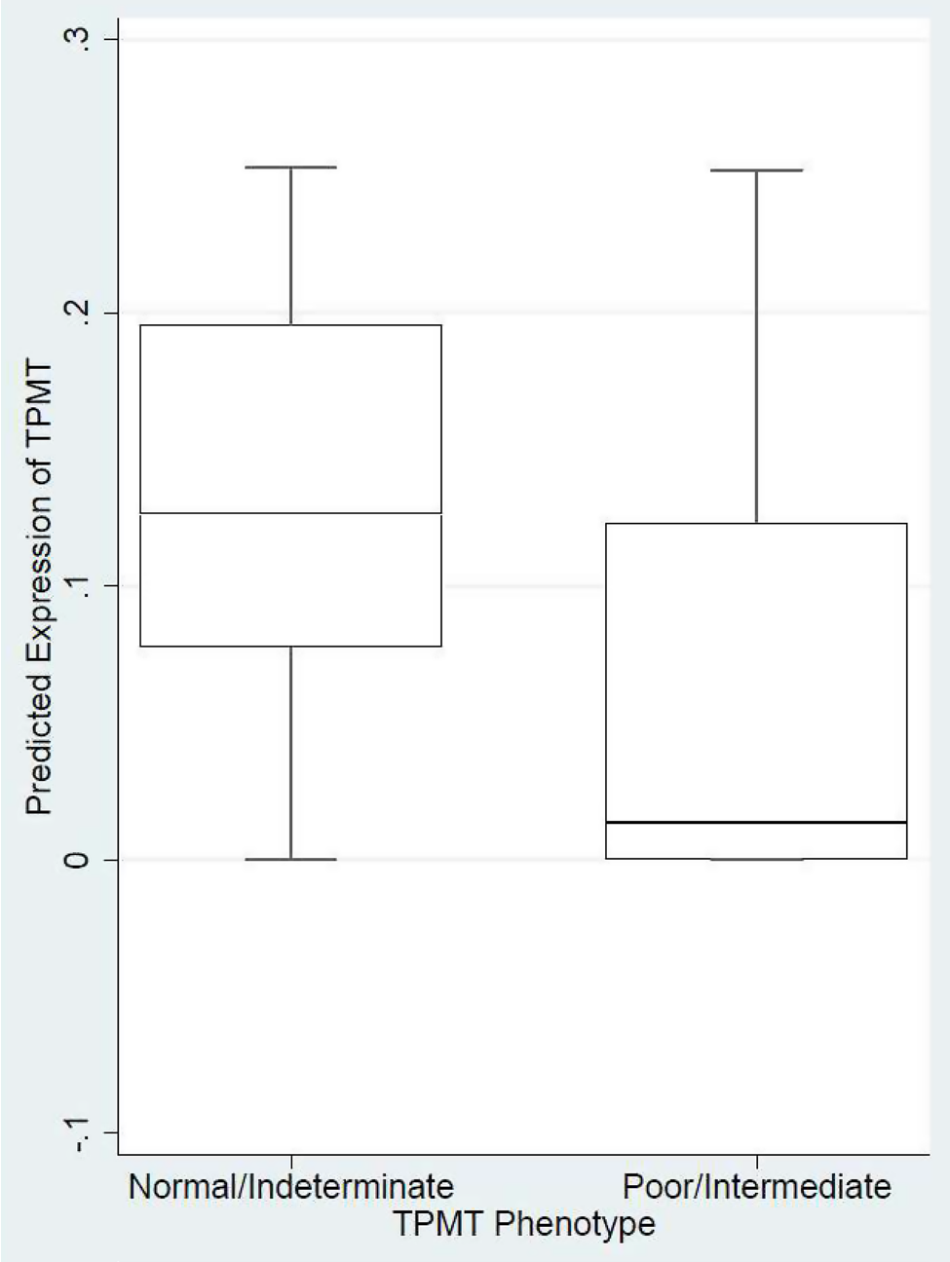
Predicted expression of *TPMT* in liver tissue by *TPMT* phenotype group

We validated the predicted expression of *NUDT15* and *TPMT* by testing the association between the predicted expression and the CPIC-defined phenotype. We found no significant association between the CPIC-defined *NUDT15* phenotype and the predicted expression of *NUDT15* in liver tissue (*p* = 0.40). There were only nine individuals for whom CPIC guidelines would classify as intermediate/poor phenotype. In addition, there was insufficient variability in the predicted expression of *NUDT15* for meaningful analysis (Figure S3). In contrast, there were 92 individuals whom CPIC guidelines would classify as *TPMT* poor/intermediate metabolizers. Using Wilcoxon rank-sum, we found that the predicted expression (unitless) of *TPMT* in liver tissue was differentially expressed between the two CPIC defined phenotype groups (*p* < 0.001; Fig. 1)

To interrogate whether the single SNP (rs2842941) in the *TPMT* liver tissue weight was an artifact of a SNP used in the CPIC guidelines, we tested to see if the PrediXcan SNP was in linkage disequilibrium with any SNP used in the CPIC guidelines on either a population basis or within the cohort and found that it was not (Table S2).

Based on PrediXcan alone (without the CPIC SNPs or the other PrediXcan SNP), we tested the association of predicted *TPMT* liver expression and the 51 phecodes representing known azathioprine side effects, using logistic regression (Table S3). Three phecodes had a pre-specified *p* < 0.10: rash (*n* = 40; *p* = 0.01), non-basal cell skin cancer (*n* = 13; *p* = 0.02), and leukopenia (*n* = 25; *p* = 0.08). For each of the phecodes, we completed chart review of all possible cases to determine if the adverse event could be attributed to azathioprine. After clinical chart review, 25 (100% of possible cases confirmed) cases of leukopenia, 12 (92%) cases of skin cancer, and 4 (10%) cases of rash were found to be attributable (as described in Methods) to azathioprine use.

We conducted the analysis of confirmed cases using predicted expression of *TPMT* as a categorical variable (tertiles) instead of a continuous. When assessed by tertile of predicted *TPMT* expression, patients in the lowest tertile, representing those with the least predicted *TPMT* expression, had higher odds of developing leukopenia (OR = 3.30, 95%CI: 1.07–10.20, *p* = 0.04) compared to patients in the highest tertile (Table 2). The results for skin cancer were not significant for the lowest tertile versus the highest tertile (OR = 0.28, 95%CI: 0.06–1.36, *p* = 0.12). The number of confirmed rash cases that were clinically attributable to azathioprine was too small for meaningful results.

**Table 2:** Association between predicted *TPMT* expression tertile and confirmed azathioprine-related side effects

	N Events	N At Risk	Unadjusted (95% CI)	Adjusted by Age (95% CI)	Adjusted by Sex (95% CI)	Adjusted by Initial Dose (95% CI)
*Leukopenia*						
Tertile 1	13	455	OR = 3.30 (1.07–10.20) *p* = 0.04	OR = 3.32 (1.08–10.28) *p* = 0.04	OR = 3.32 (1.07–10.25) *p* = 0.04	OR = 3.29 (1.06–10.16) *p* = 0.04
Tertile 2	8	456	OR = 2.00 (0.60–6.70) *p* = 0.26	OR = 2.01 (0.60–6.71) *p* = 0.26	OR = 2.01 (0.60–6.72) *p* = 0.26	OR = 2.00 (0.60–6.70) *p* = 0.26
Tertile 3	4	453	*	*	*	*
*Skin Cancer*						
Tertile 1	2	455	OR = 0.28 (0.06–1.36) *p* = 0.12	OR = 0.29 (0.06–1.39) *p* = 0.12	OR = 0.27 (0.06–1.31) *p* = 0.11	OR = 0.28 (0.06–1.34) *p* = 0.11
Tertile 2	3	456	OR = 0.42 (0.11–1.64) *p* = 0.21	OR = 0.42 (0.11–1.63) *p* = 0.21	OR = 0.41 (0.11–1.62) *p* = 0.21	OR = 0.43 (0.11–1.66) *p* = 0.22
Tertile 3	7	453	*	*	*	*

Tertile 3 (highest predicted expression) is the reference

We also assessed the ability of the predicted expression of *TPMT* to capture low WBC based on lab values. We found that the tertile with the least predicted expression of *TPMT* was associated with a WBC lab value of less than or equal to 3.0 K/uL (OR = 1.77: CI = 1.16–2.69; *p* = 0.008). In a sensitivity analysis using a WBC cutoff of 4.0, we found similar results (OR = 1.30: CI = 0.97–1.74; *p* = 0.08).

We next examined the additional cases of leukopenia captured by the predicted expression of *TPMT* when compared to metabolizer status (per CPIC guidelines). Among the 92 individuals who were *TPMT* poor/intermediate, 2 (2.17%) were cases and among the 455 patients in the lowest tertile, 13 (2.86%) were cases – this included the 2 *TPMT* poor/intermediate metabolizers.

## Discussion

In this proof-of-concept study, PrediXcan identified that a genetically predicted expression of *TPMT* in liver tissue was associated with leukopenia among new users of azathioprine. However, PrediXcan did not find significant associations between predicted *TPMT* expression and skin cancer or rash. Assessing the predicted expression may be especially helpful in identifying clinically significant genetic polymorphisms involved in drug metabolism [28], which is important for the advancement of personalized medicine. Our findings suggest that PrediXcan may be useful for interrogating the association between predicted gene expression and side effects of medications, but further research is needed to more comprehensively examine its utility.

Known variants in genes encoding enzymes involved in the metabolism of thiopurines, such as *TPMT* and *NUDT15*, have strong effects and can help us predict whether a patient will have an adverse event. [5] However, it is likely that many adverse events experienced by patients result from additional variants or the interaction of multiple variants. [6] PrediXcan, particularly when paired with the use of phecodes, offers us a high throughput method to test the association between the a single gene and multiple side effects. Indeed, given that 23 of the 25 cases of leukopenia detected by this approach had normal *TPMT* metabolizer status per current CPIC guidelines, this newly identified predicted expression may provide clinical utility if added to those guidelines. While PrediXcan combined with MASHR weights chose only one SNP, our results indicate this SNP in combination with the MASHR weights is informative.

Previous studies have demonstrated that individuals with the *TPMT* alleles that result in the inhibited metabolism of azathioprine have an increased risk of developing leukopenia while taking azathioprine, and this consensus is reflected in the CPIC guidelines for taking thiopurine drugs. [3, 5, 29] We found reduced *TPMT* predicted expression was also associated with azathioprine-induced leukopenia. The SNP rs2842941 is located in the five prime untranslated region (UTR) of *TPMT*. It has an allele frequency of rs2842941 (C > G:0.503) in European populations. [30] There are no reports of this variant in ClinVar. Given that the SNP used to predict *TPMT* expression in liver tissue was external to those used to develop CPIC guidelines, the findings suggest that the use of PrediXcan paired with phecodes may be a robust method for detecting previously unknown associations between predicted gene expression and adverse events, particularly if the impact of individual SNPs are not detectable by GWAS or are mediated by an individual’s larger genetic profile.

This study has limitations. First, GTEx models were validated in populations primarily composed of individuals with predominantly European ancestry. [31] As such, we limited this analysis to White patients. We explored the possibility of expanding the cohort to include all races to test the feasibility of applying this methodology to patients with non-European ancestry, but the numbers were too small for any meaningful assessment; there were no additional cases of skin cancer, and only four potential cases of leukopenia. Second, the cohort size limited our ability to assess *NUDT15* predicted expression. Third, the cohort size limited our power to detect significant differences in outcomes that were rare. Larger studies may provide further insight regarding the relationship between predicted expression of *TPMT* and skin cancer as well as allow the exploration of applying this methodology to patients with non-European ancestry. Fourth, our analysis was restricted to predicted expression in liver tissue, not other tissues. While this pre-specified choice seemed most appropriate given that metabolism of azathioprine is primarily hepatic, results may differ if performed using different tissues. Fifth, the phecodes may not be as sensitive for detecting side effects as directly gathering clinical data (e.g., laboratory results). Indeed, the results for rash suggest the potential difficulty of distinguishing between detecting azathioprine-associated side effects for diagnoses associated with azathioprine indications. Finally, while GTEx/PrediXcan offers opportunities to link genotypes to the transcriptome (eQTL), the emerging large-scale proteomic efforts could further enhance our ability to link genotypes to protein levels and enzyme function. The Pharma Proteomics Project has characterized the plasma proteome profiles of more than 54,000 UK Biobank participants. This resource will provide deeper insights into protein function and its contribution to adverse drug reactions.[32]

## Conclusion

In this proof-of-concept study, PrediXcan identified an association between the genetically predicted expression of *TPMT* and azathioprine-induced leukopenia. We demonstrated that the predicted expression of *TPMT* was significantly associated with leukopenia—a gene already implicated in azathioprine-related adverse effects. This finding indicates PrediXcan may be a valuable tool for researchers, demonstrating its potential to uncover associations between genetic risk factors and drug side effects. However, more research is needed to assess PrediXcan’s utility. Future research could expand on this approach by exploring the predicted expression of other genes involved in drug metabolism, potentially enabling more personalized and precise dosing recommendations.

## Supplementary material

Below is the link to the electronic supplementary material.

Supplementary Material 1.

## Data Availability

The datasets generated and/or analyzed during the current study are not publicly available because access to the data requires a DUA with VUMC. The corresponding author can be contacted to facilitate the DUA request.

## Abbreviations

CPIC: Clinical Pharmacogenetics Implementation Consortium
EHR: Electronic health record
GTEx: Genotype-tissue expression
IBD: Inflammatory bowel disease
ICD: International classification of disease
MASHR: Multivariate adaptive shrinkage in R
MEGA^EX^: Expanded Multi-Ethnic Genotyping Array
NUDT15: Nudix hydrolase 15
SLE: Systemic lupus erythematosus
SNP: Single nucleotide polymorphism
TPMT: Thiopurine-S-methyltransferase
VUMC: Vanderbilt University Medical Center
WBC: White blood cell
