# Supplementary material for: Association between genetically predicted expression of TPMT and azathioprine adverse events
Source: BMC Pharmacol Toxicol. 2026 Feb 7;27:89. doi: 10.1186/s40360-026-01093-4 (PMC13295855; doi:10.1186/s40360-026-01093-4)
Supplement: Supplementary file 1 — Supplementary Material 1. [file 40360_2026_1093_MOESM1_ESM.docx]

**SUPPLEMENT**

Table S1: Phecodes Representative of Azathioprine Side Effect Types

Figure S1: Case Adjudication Form for Potential Azathioprine Side Effects

Figure S2: Inclusion Criteria

Figure S3: Histogram of NUDT15 Predicted Liver Expression

Table S2: Available TPMT Metabolizer Phenotype SNPs and Linkage Disequilibrium with rs2842941

Table S3: Association of Predicted TPMT Expression in Liver Tissue with 50 Phecodes Representative of Azathioprine Side Effects

**Table S1: Phecodes Representative of Azathioprine Side Effect Types**

| **Azathioprine Side Effect Types** | **Grouped Phecode** | **Phecode Description** | **Component Codes** | **Component Description** |
| --- | --- | --- | --- | --- |
| Infections | 008 | Intestinal Infection | 008.5 | bacterial enteritis |
|  |  |  | 008.51 | intestinal e coli |
|  |  |  | 008.52 | intestinal infection due to C Diff |
|  |  |  | 008.6 | viral enteritis |
|  |  |  | 008.7 | intestinal infection due to protozoa |
|  | 010 | Tuberculosis | 010 | Tuberculosis |
|  | 031 | Diseases due to other mycobacteria | 031 | Diseases due to other mycobacteria |
|  | 038 | Septiciemia | 038 | Septiciemia |
|  |  |  | 038.1 | Gram negative septicemia |
|  |  |  | 038.2 | Gram positive septicemia |
|  |  |  | 038.3 | Bacteremia |
|  | 994 | Sepsis and SIRS | 994 | Sepsis and SIRS |
|  |  |  | 994.1 | SIRS |
|  |  |  | 994.2 | sepsis |
|  |  |  | 994.21 | septic shock |
|  | 041 | Bacterial Infection NOS | 041 | Bacterial Infection NOS |
|  |  |  | 041.1 | Staphylococcus infection |
|  |  |  | 041.11 | MSSA |
|  |  |  | 041.12 | MRSA |
|  |  |  | 041.2 | Streptococcus infection |
|  |  |  | 041.21 | Rheumatic fever/chorea |
|  |  |  | 041.4 | E.coli |
|  |  |  | 041.8 | H. pylori |
|  |  |  | 041.9 | infection with drug resistant microorganisms |
|  | 053 | Herpes Zoster | 053 | Herpes Zoster |
|  |  |  | 053.1 | Herpes zoster with nervous system complications |
|  | 054 | Herpes Simplex | 054 | Herpes Simplex |
|  | 070 | Viral hepatitis | 070 | Viral Hepatitis |
|  |  |  | 070.1 | Viral Hepatitis A |
|  |  |  | 070.2 | Viral Hepatitis B |
|  |  |  | 070.3 | Viral Hepatitis C |
|  | 070.4 | Chronic hepatitis | 070.4 | Chronic hepatitis |
|  | 070.9 | Hepatitis NOS | 070.9 | Hepatitis NOS |
|  | 071 | Human immunodeficiency virus [HIV] disease | 070 | HIV disease |
|  |  |  | 070.1 | HIV infection, symptomatic |
|  | 079 | Viral infection | 079 | Viral infection |
|  |  |  | 079.1 | Varicella infection |
|  |  |  | 079.2 | Infectious mononucleosis |
|  |  |  | 079.9 | viremia, NOS |
|  | 481 | Influenza | 481 | Influenza |
|  | 112 | Candidiasis | 112 | Candidiasis |
|  |  |  | 112.3 | Candidiasis of skin and nails |
|  | 117 | Mycoses | 117 | mycoses |
|  |  |  | 117.1 | histoplasmosis |
|  |  |  | 117.2 | Coccidioidomycosis |
|  |  |  | 117.3 | Blastomycotic infection |
|  |  |  | 117.4 | aspergillus |
|  | 131 | Protozoan infection | 131 | Protozoan infection |
|  | 320 | Meningitis | 320 | Meningitis |
|  | 324 | Other CNS infection and poliomyelitis | 324 | Other CNS infection and poliomyelitis |
|  | 464 | Acute sinusitis | 464 | Acute sinusitis |
|  | 480 | Pneumonia | 480.1 | bacterial pneumonia |
|  |  |  | 480.11 | Pneumococcal pneumonia |
|  |  |  | 480.12 | Pseudomonal pneumonia |
|  |  |  | 480.13 | MRSA pneumonia |
|  |  |  | 480.2 | Viral pneumonia |
|  |  |  | 480.3 | fungal pneumonia |
|  | 590 | Pyelonephritis | 590 | Pyelonephritis |
|  | 592 | Cystitis and urethritis | 592.11 | Acute cystitis |
|  | 681 | Superficial cellulitis and abscess | 681 | cellulitis or abscess |
|  |  |  | 681.1 | cellulitis fingers/toes |
|  |  |  | 681.2 | cellulitis face/neck |
|  |  |  | 681.3 | cellulitis arm/hand |
|  |  |  | 681.5 | cellulitis leg |
|  |  |  | 681.6 | cellulitis foot/toe |
|  |  |  | 681.7 | cellulitis trunk |
| Gastrointestinal Intolerance | 561 | Symptoms involving digestive system | 561 | Symptoms involving digestive system |
|  |  |  | 561.1 | Diarrhea |
|  | 785 | Abdominal pain | 785 | Abdominal pain |
|  | 789 | Nausea and vomiting | 789 | Nausea and vomiting |
|  |  |  | 789.1 | Persistent vomiting |
|  | 577 | Diseases of pancreas | 577 | Diseases of pancreas |
|  |  |  | 577.1 | Acute pancreatitis |
|  | 260.6 | Anorexia | 260.6 | Anorexia |
| Hematologic Toxicity | 284 | Aplastic Anemia | 284 | Aplastic Anemia |
|  |  |  | 284.1 | Pancytopenia |
|  |  |  | 284.2 | constitutional aplastic anemia |
|  | 288.1 | Decreased white blood cell count | 288.1 | Decreased WBC |
|  |  |  | 288.11 | Neutropenia |
|  | 287 | Purpura and other hemorrhagic conditions | 287.3 | Thrombocytopenia |
| Dermatologic Symptoms | 687.1 | Rash and other nonspecific skin eruption | 687.1 | Rash and other nonspecific skin eruption |
|  | 689 | Disorder of skin and subcutaneous tissue NOS | 689 | Disorder of skin and subcutaneous tissue NOS |
|  | 695 | Erythematous conditions | 695.1 | toxic erythema |
|  |  |  | 695.9 | unspecified erythematous condition |
|  | 698 | Pruritus and related conditions | 698 | Pruritus and related conditions |
|  | 704 | Diseases of hair and hair follicles | 704.1 | alopecia NOS |
|  | 939 | Atopic/contact dermatitis due to other or unspecified | 939 | Atopic/contact dermatitis due to other or unspecified |
|  | 947 | Urticaria | 947 | Urticaria |
| Malignancies – Skin (Basal) | 172.21 | Skin cancer - Basal | 172.21 | Basal cell |
| Malignancies – Skin (non-Basal cell) | 172 | Skin cancer - Other | 172.22 | Squamous cell |
|  |  |  | 172.3 | carcinoma in situ |
| Malignancies - Non Skin | 200 | Myeloproliferative disease | 200 | Myeloproliferative disease |
|  |  |  | 200.1 | polycythemia vera |
|  | 204 | Leukemia | 204 | leukemia |
|  |  |  | 204.1 | Lymphoid leukemia |
|  |  |  | 204.11 | Lymphoid leukemia, acute |
|  |  |  | 204.12 | Lymphoid leukemia, chronic |
|  |  |  | 204.2 | Myeloid leukemia |
|  |  |  | 204.21 | Myeloid leukemia, acute |
|  |  |  | 204.22 | Myeloid leukemia, chronic |
|  |  |  | 204.3 | Monocytic leukemia |
| Hepatotoxicity | 573.4 | Acute and subacute necrosis of liver | 573.4 | Acute and subacute necrosis of liver |
|  | 573.6 | Nonspecific elevation of levels of transaminase or lactic acid dehydrogenase [LDH] | 573.6 | Nonspecific elevation of levels of transaminase or lactic acid dehydrogenase [LDH] |
|  | 573.7 | Abnormal results of function study of liver | 573.7 | Abnormal results of function study of liver |
|  | 573.9 | Abnormal serum enzyme levels | 573.9 | Abnormal serum enzyme levels |
| Constitutional Symptoms | 745 | Pain in joint | 745 | Pain in joint |
|  | 783 | Fever of unknown origin | 783 | Fever of unknown origin |
|  | 798 | Malaise and fatigue | 798 | Malaise and fatigue |
| Pulmonary Symptoms | 504 | Other alveolar and parietoalveolar pneumonopathy | 504.1 | idiopathic fibrosing alveolitis |

**Figure S1: Case Adjudication Form for Potential Azathioprine Side Effects**


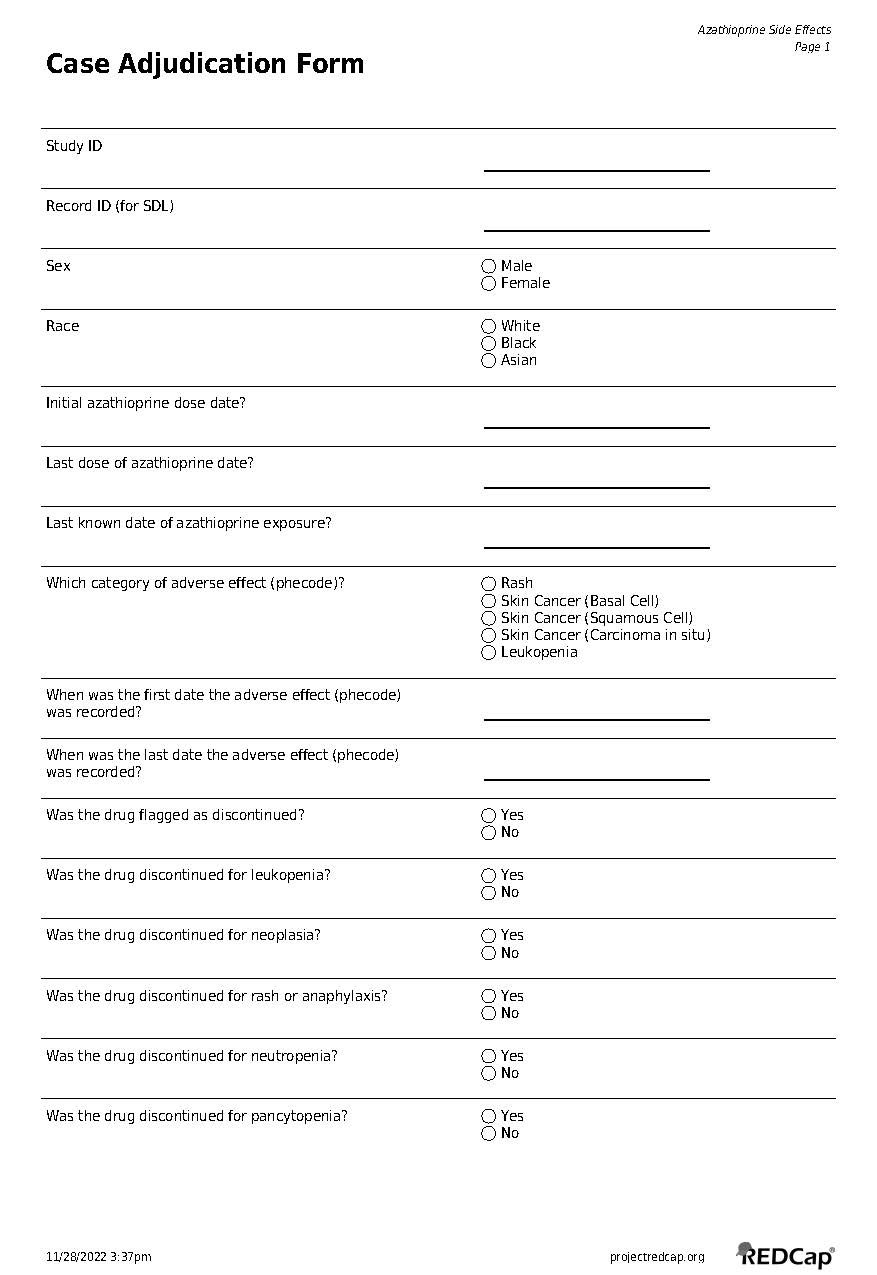


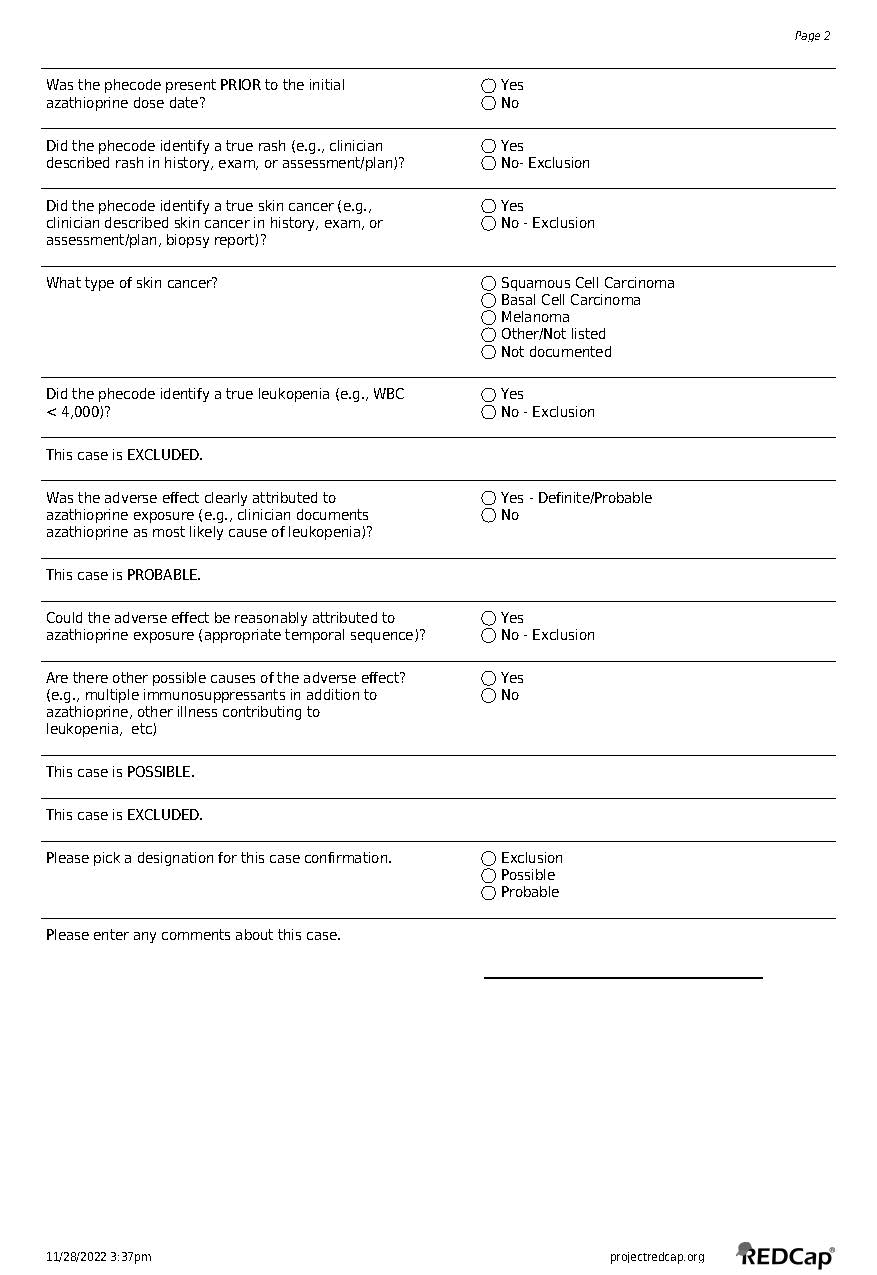


**Figure S2: Inclusion Criteria**

**
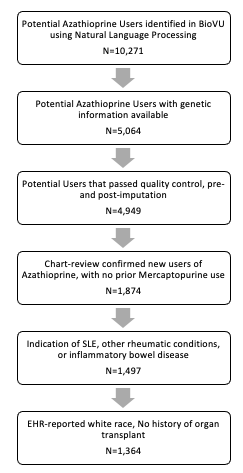
**

**Figure S3: Histogram of NUDT15 Predicted Liver Expression**


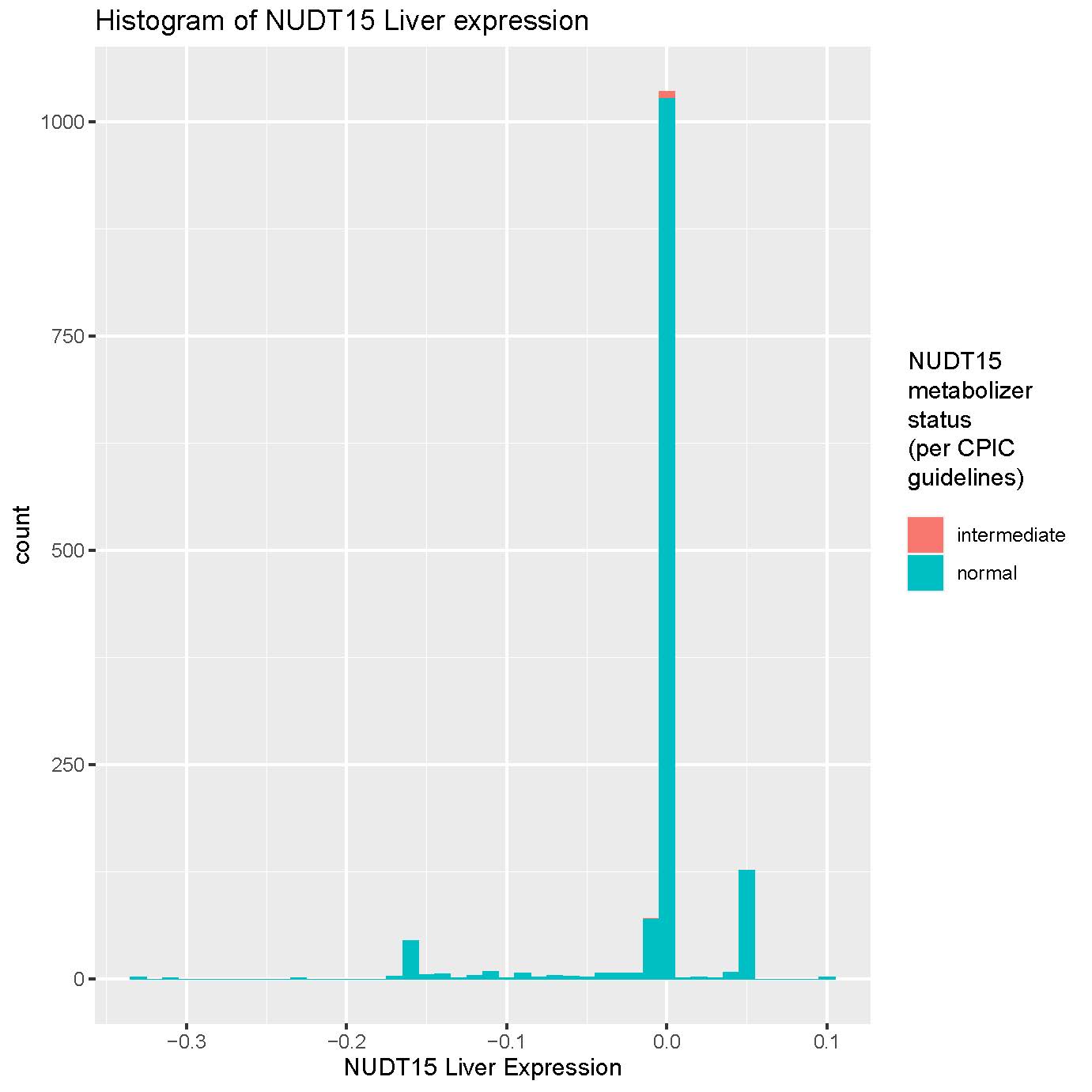


**Table S2: Available TPMT Metabolizer Phenotype SNPs and Linkage Disequilibrium with rs2842941**

| **SNP*** | **RSID** | **rs2842941 R^2^ (population-basis)**** | **rs2842941 R^2^**  **(cohort-basis)** |
| --- | --- | --- | --- |
| 6:18130918:T:C | rs1142345 | 0.031 | 0.044 |
| 6:18130993:C:T | rs56161402 | <0.001 | 0.001 |
| 6:18134078:C:A | rs6921269 | <0.001 | 0.002 |
| 6:18139228:C:T | rs1800460 | 0.030 | 0.034 |
| 6:18139941:G:A | rs200220210 | 0.001 | 0.001 |
| 6:18143837:T:G | rs151149760 | 0.001 | 0.001 |
| 6:18143955:C:G | rs1800462 | 0.006 | 0.001 |

*These SNPs account for 98.89% of *TPMT* diplotypes among patients of European descent (CPIC: <https://cpicpgx.org/guidelines/guideline-for-thiopurines-and-tpmt/>) & PharmGBK: <https://www.pharmgkb.org/page/tpmtRefMaterials>)

**Calculated from LDlink (<https://ldlink.nci.nih.gov/>)

**Table S3: Association of Predicted TPMT Expression in Liver Tissue with 50 Phecodes Representative of Azathioprine Side Effects**

| **Grouped Phecode** | **Phecode Description** | **N cases** | **N controls** | **Odds Ratio** | **p-value** |
| --- | --- | --- | --- | --- | --- |
| 687.1 | Rash and other nonspecific skin eruption | 40 | 1152 | 0.947 | 0.008 |
| 172 | Skin cancer - Other | 13 | 1236 | 1.081 | 0.035 |
| 288.1 | Decreased white blood cell count | 25 | 1208 | 0.957 | 0.079 |
| 689 | Disorder of skin and subcutaneous tissue NOS | 10 | 1216 | 1.054 | 0.188 |
| 939 | Atopic/contact dermatitis due to other or unspecified | 81 | 1130 | 0.982 | 0.199 |
| 172.21 | Basal cell carcinoma | 10 | 1236 | 1.052 | 0.203 |
| 798 | Malaise and fatigue | 222 | 910 | 0.989 | 0.206 |
| 287 | Purpura and other hemorrhagic conditions | 24 | 1212 | 1.032 | 0.214 |
| 573.4 | Acute and subacute necrosis of liver | 5 | 1235 | 0.946 | 0.332 |
| 573.6 | Nonspecific elevation of levels of transaminase or lactic acid dehydrogenase [LDH] | 19 | 1215 | 1.027 | 0.342 |
| 038 | Septicemia | 37 | 1207 | 1.017 | 0.390 |
| 783 | Fever of unknown origin | 74 | 1123 | 1.012 | 0.394 |
| 504 | Other alveolar and parietoalveolar pneumonopathy | 23 | 1223 | 0.979 | 0.406 |
| 561 | Symptoms involving digestive system | 114 | 1067 | 1.010 | 0.422 |
| 041 | Bacterial infection NOS | 45 | 1202 | 1.014 | 0.461 |
| 681 | Superficial cellulitis and abscess | 59 | 1184 | 1.011 | 0.487 |
| 573.7 | Abnormal results of function study of liver | 51 | 1159 | 1.012 | 0.498 |
| 053 | Herpes zoster | 12 | 1228 | 0.976 | 0.498 |
| 008 | Intestinal infection | 21 | 1228 | 1.017 | 0.527 |
| 054 | Herpes simplex | 13 | 1226 | 0.979 | 0.539 |
| 789 | Nausea and vomiting | 90 | 1102 | 1.008 | 0.551 |
| 704 | Diseases of hair and hair follicles | 8 | 1235 | 0.975 | 0.558 |
| 284 | Aplastic anemia | 17 | 1235 | 1.015 | 0.605 |
| 577 | Diseases of pancreas | 17 | 1229 | 1.012 | 0.685 |
| 070.9 | Hepatitis NOS | 14 | 1219 | 1.013 | 0.688 |
| 070.4 | Chronic hepatitis | 144 | 1089 | 0.996 | 0.692 |
| 785 | Abdominal pain | 160 | 998 | 1.004 | 0.729 |
| 480 | Pneumonia | 11 | 1234 | 1.012 | 0.752 |
| 947 | Urticaria | 7 | 1238 | 1.012 | 0.789 |
| 117 | Mycoses | 6 | 1245 | 1.013 | 0.799 |
| 071 | Human immunodeficiency virus [HIV] disease | 102 | 1137 | 0.997 | 0.800 |
| 745 | Pain in joint | 172 | 1004 | 0.998 | 0.847 |
| 112 | Candidiasis | 24 | 1203 | 1.005 | 0.854 |
| 079 | Viral infection | 15 | 1222 | 1.006 | 0.857 |
| 464 | Acute sinusitis | 27 | 1188 | 1.004 | 0.861 |
| 592 | Cystitis and urethritis | 5 | 1243 | 1.006 | 0.906 |
| 994 | Sepsis and SIRS | 42 | 1211 | 0.998 | 0.906 |
| 573.9 | Abnormal serum enzyme levels | 21 | 1212 | 1.002 | 0.927 |
| 590 | Pyelonephritis | 7 | 1240 | 1.003 | 0.950 |
| 070 | Viral hepatitis | 105 | 1137 | 1.001 | 0.963 |
| 320 | Meningitis | 3 | 1247 | * | * |
| 698 | Pruritus and related conditions | 3 | 1230 | * | * |
| 481 | Influenza | 3 | 1239 | * | * |
| 695 | Erythematous conditions | 2 | 1235 | * | * |
| 010 | Tuberculosis | 2 | 1251 | * | * |
| 260.6 | Anorexia | 2 | 1244 | * | * |
| 131 | Protozoan infection | ** | ** | * | * |
| 324 | Other CNS infection and poliomyelitis | 2 | 1249 | * | * |
| 031 | Diseases due to other mycobacteria | ** | ** | * | * |
| 200 | Myeloproliferative disease | ** | ** | * | * |
| 204 | Leukemia | 1 | 1252 | * | * |

*Insufficient cases for results

**No cases
